# Estimation of Motor Impairment and Usage of Upper Extremities during Daily Living Activities in Poststroke Hemiparesis Patients by Observation of Time Required to Accomplish Hand Dexterity Tasks

**DOI:** 10.1155/2019/9471921

**Published:** 2019-11-07

**Authors:** Tomoko Tanaka, Toyohiro Hamaguchi, Makoto Suzuki, Daigo Sakamoto, Junpei Shikano, Naoki Nakaya, Masahiro Abo

**Affiliations:** ^1^Department of Rehabilitation Medicine, The Jikei University School of Medicine, Tokyo, Japan; ^2^Department of Rehabilitation, Graduate School of Health Sciences, Saitama Prefectural University, Saitama, Japan; ^3^Department of Rehabilitation, Kyoto-Ohara Memorial Hospital, Kyoto, Japan

## Abstract

**Aim:**

This study evaluated whether specific actual performance could accurately predict body function levels and upper limb use in the real-life functioning of poststroke hemiparesis patients to aid in choosing the most appropriate rehabilitation exercises.

**Methods:**

We measured the time taken for poststroke patients to move small objects with the paralyzed hand and investigated how the measurement could estimate upper extremity motor impairment and hand usage during activities of daily living (ADL). We examined 86 stroke patients (age 66 ± 16 years) whose upper extremity motor paralysis was measured using the Fugl-Meyer assessment (FMA) and Southampton Hand Assessment Procedure (SHAP), and patient-reported ADL was investigated using the Jikei Assessment Scale for Motor Impairment in Daily Living (JASMID). To identify the time required to perform each SHAP item, we employed a linear regression analysis. The prediction formula was used in the linear regression analysis, and the coefficient of determination (*R*^2^) was applied to compare each component item score that was obtained with the predicted values derived from the linear regression analysis.

**Results:**

The most easily accomplished task was Heavy Power in the SHAP. The *R*^2^ between the SHAP Heavy Power item score and the FMA scores was moderate (*R*^2^ = 0.344, *P* < 0.0001), whereas the *R*^2^ with the JASMID score was low (*R*^2^ = 0.126, *P* < 0.001).

**Conclusions:**

By measuring the time it takes for poststroke hemiparesis patients to hold and move an object, we developed a prediction formula for upper extremity motor function and hand dexterity.

## 1. Introduction

A high rate of upper extremity paralysis remains in stroke patients [1, 2]. Repetitive exercises involving the hands have been included in rehabilitation programs for patients with upper body paralysis, as they are commonly used during ADLs [3, 4]. These repetitive exercises in which the patient actively uses their upper extremities during ADLs have been shown to be effective for poststroke hemiparesis recovery [5, 6].

When patients with poststroke upper extremity paralysis perform ADLs, the therapist measures the motor function of the patient's upper extremities to estimate the effect of treatment intervention [7], and the patient can also estimate his or her ability to use their paralyzed upper extremities during ADLs [8]. Methods for assessing the motor function of the fingers in stroke patients include the Brunnstrom Stage (BRS) [9] and Fugl-Meyer Assessment (FMA) [10]. The Wolf Motor Function Test (WMF) [11] and the Southampton Hand Assessment Procedure (SHAP) [12] are lab-based measures of activity. Methods for assessing hand usage in stroke patients while performing ADLs (how much and how well the subject uses their most-affected arm outside of the laboratory setting) include the Motor Activity Log (MAL) [13, 14] and the Jikei Assessment Scale for Motor Impairment in Daily Living (JASMID) [15]. The MAL includes 14 questions, and the JASMID includes 20 questions regarding how much and how well the patient uses their paretic arm to complete ADLs. These assessment methods for hand dexterity include many questions, and they can be time-consuming for patients and clinicians [16]. If these assessments could be made more efficient with the use of fewer items, while still accurately estimating the function and extent of upper extremity usage, then doctors may be able to spend more time on planning and implementing effective rehabilitation programs.

Therefore, we conducted a cross-sectional study to predict the levels of body function for upper limb paresis and self-reported upper limb use in real-life activities by measuring the actual specific activity performance. Based on the background information on impairments and disabilities relevant to hemiparesis after stroke, we hypothesized that (a) some hand dexterity tasks will need a shorter time to carry out than others and (b) upper extremity motor function and hand functioning satisfaction during ADLs can be estimated by measuring the time required for patients to hold and move an object.

## 2. Materials and Methods

### 2.1. Participants

Patients with a poststroke upper extremity paralysis who were examined at the Department of Rehabilitation of the Jikei University Hospital and Kyoto-Ohara Memorial Hospital between January 8, 2015, and March 15, 2017, were included in this study. The inclusion criteria were (1) a motor function assessment score of at least 20 on the FMA of the Upper Extremity (FMA-UE) for the ability to control objects with one hand [17] and (2) no cognitive impairment. Informed consent was obtained, and 95 patients who agreed to participate in the study were enrolled. Finally, 86 patients who completed the assessment (mean age 66 ± 16 years; interquartile range (IQR): 51–74 years; females 33 [34.7%]) were included in the analysis. Lesions were diagnosed using head magnetic resonance imaging, and the findings showed that 48 patients had a cerebral infarction, 28 had a cerebral hemorrhage, seven had a subarachnoid hemorrhage, one had moyamoya disease, one had a cerebral artery malformation, and one had a cerebral trauma (Table 1).

There were 35 patients with damaged right hemispheres and 51 with damaged left hemispheres. The date of assessment was 4 ± 45 (IQR: 2–10) months after onset. This study was approved by the Institutional Review Board at the Jikei University Hospital (No. 26–138).

### 2.2. Tools

Poststroke hemiparesis was assessed by occupational therapists using the FMA [10]. The FMA is designed to assess motor functioning, balance, sensation, and joint functioning in patients with poststroke hemiparesis [10, 18]. It is applied clinically and in research to determine disease severity, describe motor recovery, and to plan and evaluate treatment. FMA scoring is based on a direct observation of performance. Scale items are scored based on the ability to complete each item using a 3-point ordinal scale where 0 = cannot perform, 1 = can perform partially, and 2 = can perform fully. The boundary FMA-UE score between severe and moderate impairment was defined as 20 points and that between moderate and mild impairment was defined as 49 points [17].

Hand function was assessed using the SHAP [12]. The SHAP is a clinically validated hand function test, and it is made up of six abstract objects and 14 ADLs. Each task in the SHAP is timed by the participant, so there is no interference or reliability on the reaction times of the observer or clinician [19]. Healthy subjects accomplish each Abstract Object Task in a few seconds in the SHAP. To estimate upper extremity function in hemiplegic patients after stroke, SHAP items were used to measure the time taken to hold and move small objects on the surface of a table. Each patient underwent three trials of each SHAP item, and the mean duration of the three trials was calculated.

The JASMID [15], which is a patient-reported measure, was adopted to investigate the usage of the upper extremities during ADLs. JASMID scores assess the usage frequency and quality of movements on a 5-point scale (0: unused; 3: mild limitation; 5: no limitation) for all 20 ADL items that are related to upper extremity exercises, and a score is calculated for each item.

### 2.3. Data Analyses

To identify the time required to perform the Abstract Object Tasks in the SHAP, the total score for all items, as well as the score for each of the component items, was analyzed using linear regression:

(1) $$\begin{array}{c} f\left(x\right)=\alpha +\beta x, \end{array}$$

where *f* (*x*) is each component item score, *x* is the total Abstract Object Tasks Score or Activities of Daily Living Score, *α* is the function constant, and *β* is the unstandardized regression coefficient. In linear regression, more difficult items shift the line to the left and have a steeper slope, whereas easier items shift the line to the right and have a gentler slope. Moreover, to clarify the difficult items, predicted scores were calculated for each item using the time required to carry out the total item (150 seconds) in the linear regression function [20]. To assess the applicability of linear regression, the determination coefficient (*R*^2^) was used to compare each component item score that was obtained with the predicted values derived from the linear regression analysis. In this study, “difficult item” was defined by the time required to perform each SHAP item.

In addition, to determine the association between the severity of hand clumsiness and impairments and disabilities relevant to arm function, the coefficient of determination (*R*^2^) was also determined using linear regression (equation (1)), where *f* (*x*) was the FMA or JASMID score, *x* was the score of the easiest item of the Abstract Object Tasks of the SHAP, *α* was the function constant, and *β* was the unstandardized regression coefficient. *R*^2^ is perhaps the most widely used measure of fitting used in linear regression modeling [21]. This study chose a rule of thumb for an acceptable *R*^2^ with 0.75, 0.50, and 0.2 described as substantial, moderate, and weak, respectively [22]. A *P* value of <0.05 was considered statistically significant. All analyses were performed with *R* 3.4.0 software (R Foundation for Statistical Computing, Vienna, Austria).

## 3. Results

Among the 86 subjects, the mean upper extremity motor score of the FMA was 58 (Table 1). All timed task results on the SHAP are represented in [Supplementary-material supplementary-material-1]. The normalized SHAP scores on the six prehensile patterns were spherical = 74, power = 55, tip = 65, tripod = 43, lateral = 73, extension = 74, and index of the function = 64. The mean JASMID scores were 84 for frequency and 76 for quality (Table 1).

**Figure 1:**
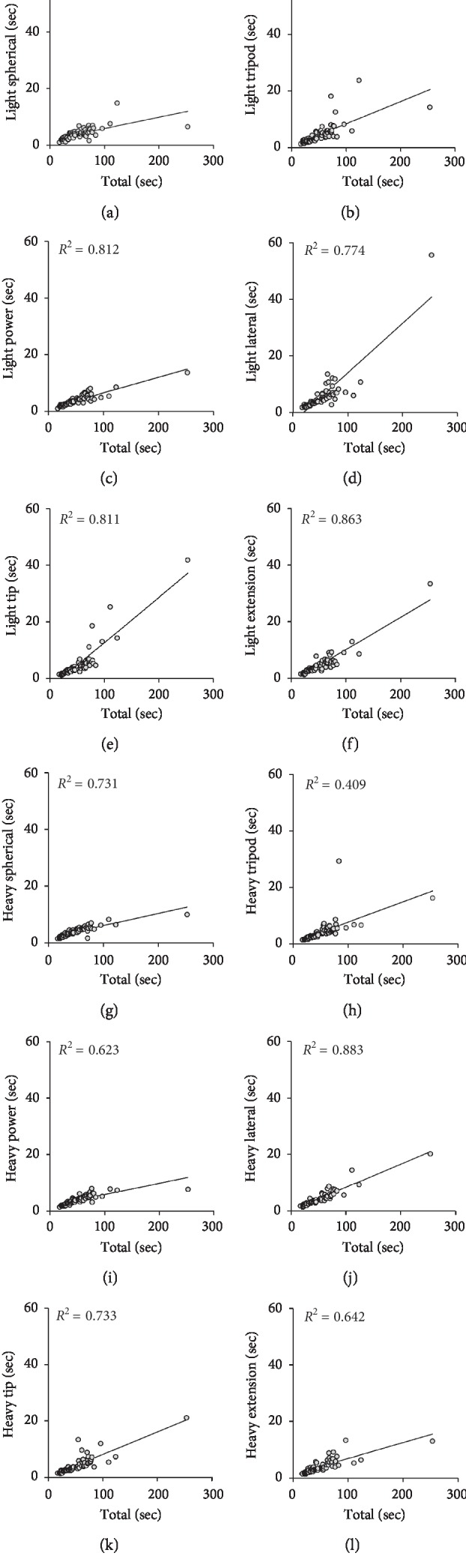
Scatter plots showing the relationship between the total times required to complete the SHAP in the Abstract Object Tasks and the component items. Light Spherical (a), Light Tripod (b), Light Power (c), Light Lateral (d), Light Tip (e), Light Extension (f), Heavy Spherical (g), Heavy Tripod (h), Heavy Power (i), Heavy Lateral (j), Heavy Tip (k), and Heavy Extension (l). SHAP: Southampton Hand Assessment Procedure.

The relationship between single component item scores and the total component item scores on the SHAP, as calculated using the linear regression modeling of the dataset, is shown in Figure 1 and Table 2. The *R*^2^ was calculated to compare the actual data and predicted values and to determine if the modeling formula accurately predicted the measured component item scores. The *R*^2^ between the actual and approximated component item scores was moderate to high, based on the linear regression model (Abstract Object Tasks: *R*^2^ = 0.40–0.88, *P* < 0.0001, Table 2).

Therefore, the 150 seconds of the SHAP total item score derived from the linear regression analysis [20] were compared for different component items ([Supplementary-material supplementary-material-1]). The SHAP Abstract Object Tasks scores for Heavy Power, Light Spherical, and Heavy Spherical were low in a stepwise fashion, whereas those for Light Lateral, Light Tip, and Light Extension were high in a stepwise fashion. Among the Abstract Object Tasks items, Heavy Power was the easiest component to perform, with Heavy Spherical being the most difficult.

**Figure 2:**
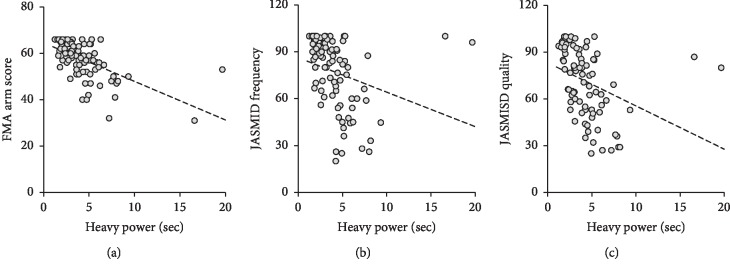
Scatter plots showing the relationship between the times required to complete the SHAP Heavy Power item and other scores: FMA upper extremity score (a), JASMID frequency score (b), and JASMID quality score (c).

The *R*^2^ between the SHAP Heavy Power item score and FMA scores was moderate, whereas the *R*^2^ between the SHAP Heavy Power item score and JASMID scores was low (Figure 2 and Table 3).

## 4. Discussion

This study measured the time required for patients with poststroke hemiparesis to grasp and move an object during the heavy power lift item of the SHAP. We attempted to estimate the extent of motor paralysis and hand ability during ADLs using this value. Our results indicated that (a) some hand dexterity tasks were easier to accomplish than others and (b) the severity of hand clumsiness correlated with arm paresis and use of the affected arm during ADLs. The coefficient of determination of the required time for each individual SHAP item, as well as the coefficient of determination of the total required time for all items, was high. Furthermore, we developed a formula to estimate the FMA and JASMID scores according to the time required for stroke patients to complete the Heavy Power task on the SHAP. The Light Lateral task of the SHAP was considered the most difficult, while the Heavy Power task was the least difficult. This led us to the observation that the score for each criterion could be estimated from the time taken to complete the Heavy Power task.

The FMA is widely used in clinical trials to quantify motor deficits after a stroke, and it takes approximately 60 min to employ the entire FMA [16]. The average length of time to complete the motor, sensation, and balance subsections of the FMA has been reported to range from 34 to 110 minutes, with a mean duration time of 58 minutes [16]. Thus, a 12-item short form of the FMA was developed, and items were retained based on how well they related to the BRS, with the time required to perform each SHAP item assessed via Rasch analysis [23].

The Heavy Power item from the SHAP measures the time required for patients to hold a metal cylinder (height = 10 cm, diameter = 3 cm, weight = 187 g) and move it by approximately 5 cm [24]. This maneuvering of objects can be completed in approximately 2 seconds by people without motor dysfunction [12]. Heavy Power was one of the measurement items on the SHAP that the subjects who had completed all items of the SHAP within 100 seconds could complete most quickly, and it also had the highest number of subjects who completed it. In addition, stroke patients in this study were able to perform the SHAP Heavy Power task in 3.6 seconds (IQR 2.6–5.8) (see [Supplementary-material supplementary-material-1]). The short amount of time taken to estimate the motor paralysis of the upper extremity in stroke patients using our procedure will benefit clinicians and patients; more time can be spent determining an appropriate treatment program. An inertial sensor can be attached during performance of a SHAP item to measure the position information and movement time accurately. Using the results of this study, it may be possible to estimate the degree of paralysis of the upper extremity using this sensor information. When a new metric is created with a measurement method like the SHAP's Heavy Power, it takes only a few minutes, even when the patient is given an explanation and three measurements are taken. The results of this study indicated that the time spent on Heavy Power could be substituted as an assessment of the upper extremities, in conjunction with the FMA motor score. Therefore, an approximate estimation of the upper extremity function in poststroke hemiparesis patients is considered possible by observing the time taken to hold and move a small object on the surface of a table.

The relationship between the levels of body function for upper limb paresis, actual activity performance of the paretic upper limb, and self-reported upper limb use in real-life activity is an important issue in stroke rehabilitation. Making prognostic predictions is a critical issue so that interventions that promote the recovery of the upper extremity motor function are conducted promptly in poststroke hemiparesis patients [25]. There are reports on the prediction of recovery of upper extremity motor function using the FMA [26]. Using a formula, the assessment value of the FMA can be estimated by measuring the time taken for a patient to hold and move an object with their hand. Contrarily, the frequency and state of upper extremity usage during ADLs were poorly correlated with the time taken to complete the Heavy Power task. The upper extremity motor function and the activity capacity are associated with a self-perceived ability in people with paresis after stroke (explained variances of 59% for FMA-UE and 56% UE for activity capacity: Action Research Arm Test (ARAT), *P* < 0.001) [27]. The JASMID is a patient-reported measure, and it serves as a crude proxy for the actual amount and quality of upper extremity use [15]. Patients' hands are required variously in ADLs, depending on the task. Thus, there were differences between ARAT results, as a test of the upper extremity performance, and the results of this study. Self-reported assessments are developed to use for clinical goal setting [27, 28]. More studies are needed to predict the state of the patient's objective and subjective ADLs.

The limitations of this study include its small sample size. However, due to the requirements of the study and follow-up, we only included patients with isolated motor deficits or minimal nonmotor deficits, such as aphasia and neglect (i.e., stroke patients with cognitive impairments were excluded). Thus, our study may have involved selection bias toward patients with an isolated hemiparesis. Future studies should examine whether a proportional recovery is maintained in patients with cognitive impairment or is modulated by additional nonmotor deficits [29]. A stratified analysis may provide more accurate prediction of upper extremity motor function based on severity of motor paralysis of the upper extremity and cognitive functioning. However, the coefficients for the FMA score and the frequency and quality scores of the JASMID were 0.344, 0.076, and 0.126, respectively. Clinically, upper extremity movements may be affected by pain [30], dementia [31, 32], and depressive symptoms [33, 34]. Including these factors can increase the accuracy of the model. There is a need to build models that include these factors. The present equation and coefficient of determination can be applied only to patients like those in this study. The patients in this study had impairments of mild to moderate severity (Brunnstrom Stage 4–6). The purpose of this study was to estimate the severity of motor paralysis by measuring the time required to grasp and move small items by hand. We did not intend the results to be applicable to patients with severe motor paralysis. On the other hand, we did not analyze other factors that may have had an effect on the results, such as finger contracture [35, 36], osteoarthritis, and pain [30]. Therefore, the fact that these factors may have had an effect on the equation and the coefficient of determination in this study cannot be denied. Because the FMA can assess recovery in stroke hemiplegic patients, the score is also used to set treatment goals [16, 18, 37]. The FMA consists of a 33-item upper extremity subscale; however, due to the long administration time necessary for the FMA, a short, widely accepted version was developed for daily clinical use [23]. Subitems of the FMA such as shoulder, elbow, forearm, wrist, finger, and coordinated movements were not used in this study. Each FMA subitem can be evaluated quickly by measuring the time taken to move small objects, and the results can be used to establish patient goals. Future studies should involve a larger patient population and include an analysis of subitems.

The predicted prognosis of motor function recovery of the upper extremities has been neurologically proven to differ when the prediction is made during the acute phase or made during other phases [38]. Future studies should be conducted using a sufficient sample size to determine whether the prediction formula obtained from this study is applicable for patients in the acute phase or for those where time has passed since the occurrence of the stroke. Furthermore, the motor paralysis in the subjects of this study was of stage 1–3 on the Brunnstrom Recovery Stage, and we did not include severe cases.

In conclusion, a prediction formula for the upper extremity motor function and hand dexterity in patients with poststroke hemiparesis was developed based on the findings of the present study. This formula can be used to estimate upper extremity motor function and applied to new low-cost wearable technology (inertial sensors) by substituting the time it takes for these patients to hold and move a small object.

## Figures and Tables

**Table 1:** Descriptive summary of paraplegic participants.

Descriptor	Data
No. of participants	86
Sex (F : M)	33 : 53
Age	66 ± 16
Paralyzed side (R : L)	51 : 35
Dominant hand (R : L : bilateral)	80 : 5 : 1
Diagnosis (infarction : hemorrhage : SHA : other)	48 : 28 : 7 : 3
FMA motor score	
Upper extremity	58 (52–64)
SHAP score^†^	
Spherical	74 (53–87)
Power	55 (39–83)
Tip	65 (40–86)
Tripod	43 (23–76)
Lateral	73 (37–90)
Extension	74 (55–89)
Index of function	64 (44–85)
JASMID	
Frequency	84 (60–96)
Quality	76 (53–92)

SHA: subarachnoid hemorrhage; other included diseases such as moyamoya disease, cerebral artery malformation, and head trauma. JASMID: Jikei Assessment Scale for Motor Impairment in Daily Living; FMA: Fugl-Meyer assessment. Data were mean and standard deviation or interquartile range (IQR). ^†^Using the method described by Light et al. [12], times can then be normalized to 100, and each of the 26 tasks was classified within one of the six prehensile patterns.

**Table 2:** Relationships between single component item scores and total item scores of SHAP.

	*α*	*β*	*R* ^2^	*P*
Abstract object tasks				
Spherical				
Light	10.24	10.79	0.437	<0.0001
Heavy	−14.59	17.01	0.731	<0.0001
Tripod				
Light	21.14	6.42	0.520	<0.0001
Heavy	27.50	5.64	0.409	<0.0001
Power				
Light	−4.63	14.79	0.812	<0.0001
Heavy	−6.88	15.61	0.623	<0.0001
Lateral				
Light	27.11	4.43	0.774	<0.0001
Heavy	3.321	10.73	0.883	<0.0001
Tip				
Light	26.54	5.08	0.811	<0.0001
Heavy	13.34	9.13	0.733	<0.0001
Extension				
Light	17.95	7.48	0.863	<0.0001
Heavy	6.74	11.19	0.642	<0.0001

SHAP, Southampton Hand Assessment Procedure. The coefficient of determination (*R*^2^) was used in linear regression analysis: *f* (*x*) = *α* + *β* (*x*). *f* (*x*): the component item score; *x*: the total item score; *α*: constant of the function; *β*: unstandardized regression coefficient.

**Table 3:** Relationships between SHAP Heavy Power item score and FMA or JASMID score.

	*α*	*β*	*R* ^2^	*P*
FMA				
Upper extremity, motor score	64.55	−1.66	0.344	<0.0001
JASMID				
Frequency	86.26	−2.21	0.076	0.010
Quality	83.28	−2.77	0.126	0.001

SHAP: Southampton Hand Assessment Procedure, FMA: Fugl-Meyer Assessment, JASMID: Jikei Assessment Scale for Motor Impairment in Daily Living. The coefficient of determination (*R*^2^) was used in the linear regression analysis: *f* (*x*) = *α* + *β* (*x*). *f* (*x*): the SHAP Heavy Power item score; *x*: BR, FMA, or JASMID score; *α*: function constant; *β*: unstandardized regression coefficient.

## Data Availability

The kinematics data used to support the findings of this study are available from the corresponding author upon request.

## Supplementary Materials

Supplementary Materials Table S1: mean times of the SHAP in the normative sample and derived from participants in this study. Figure S1: values of the component items according to 150 seconds of the total of all SHAP items.
