# Supplementary material for: Estimation of Motor Impairment and Usage of Upper Extremities during Daily Living Activities in Poststroke Hemiparesis Patients by Observation of Time Required to Accomplish Hand Dexterity Tasks
Source: Biomed Res Int. 2019 Nov 7;2019:9471921. doi: 10.1155/2019/9471921 (PMC6885294; doi:10.1155/2019/9471921)
Supplement: Supplementary Materials — Table S1: mean times of the SHAP in the normative sample and derived from participants in this study. Figure S1: values of the component items according to 150 seconds of the total of all SHAP items. [file 9471921.f1.pdf]

Table S1. Mean times of the SHAP in the normative sample and derived from participants in this study.

**Abstract Object Tasks**		Normative data[†] (n = 24)	Hemiplegic data (present study, n = 86)	
		Time (s)	Time (s)	IQR (s)
Spherical	Light	1.6	3.7	2.2–4.8
	Heavy	1.8	3.8	2.5–5.8
Tripod	Light	1.7	3.8	2.5–5.5
	Heavy	1.6	3.9	2.6–6.5
Power	Light	1.8	3.5	2.3–5.3
	Heavy	1.8	3.6	2.6–5.8
Lateral	Light	1.8	3.8	2.7–5.0
	Heavy	1.8	3.7	2.4–5.4
Tip	Light	1.6	3.9	2.5–5.2
	Heavy	1.6	3.9	2.6–5.4
Extension	Light	1.8	3.4	2.3–5.3
	Heavy	1.7	3.5	2.4–5.4

SHAP: Southampton Hand Assessment Procedure, IQR: interquartile range.

[†]Light CM, Chappell PH, Kyberd PJ. Establishing a standardised clinical assessment tool for pathologic and prosthetic hand function: normative data, reliability and validity. *Arch Phys Med Rehabil*. 2002; 83: 776-783.

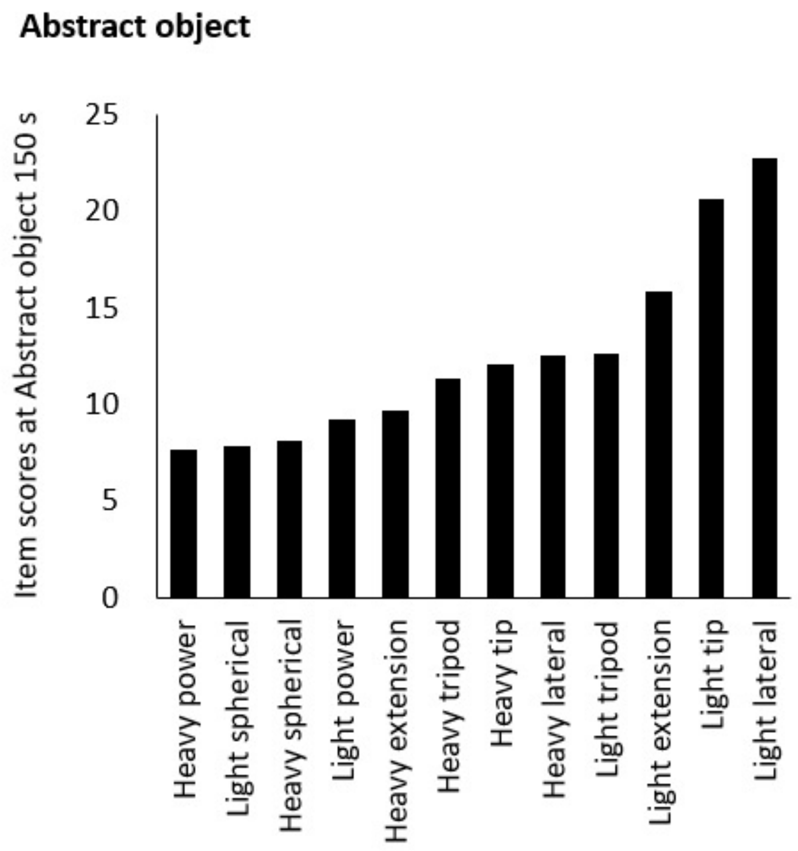

Fig S1. Values of the component items according to 150 seconds of the total of all SHAP items.

Values indicate the degree of difficulty for each component item. The Heavy Power item was the easiest item to perform and the Heavy Spherical was the most difficult item to perform in the Abstract Object Tasks test.

SHAP: Southampton Hand Assessment Procedure.
